# Experiencing Complications After Metabolic and Bariatric Surgeries is a Risk Factor for Postoperative Emergency Department Admissions: a Retrospective Cohort Study

**DOI:** 10.1007/s11695-025-07710-1

**Published:** 2025-02-01

**Authors:** Şükrü Salih Toprak, Hatice Toprak, Fulya Köse

**Affiliations:** Karamanoğlu Mehmetbey University, Karaman, Turkey

**Keywords:** Bariatric surgeries, Emergency department visits, Major complication rate, Classified complications

## Abstract

**Background:**

Emergency department admissions significantly burden hospital staff and countries’ health system. Studies are encouraged for effective and correct utilization of emergency departments. Rational management of obesity-related medical problems and postoperative complications may reduce emergency department visits. This study aimed to determine the rates, characteristics, and antecedents of emergency room admissions after metabolic and bariatric surgeries (MBSs) performed in our hospital. According to our hypothesis, experiencing postoperative complications is the most common reason for emergency department admissions.

**Methods:**

The study was designed as a single-center, retrospective, cohort study. Metabolic and bariatric surgeries performed in our hospital between June 2021 and June 2023 were evaluated. Clavien Dindo Classification was used to classify complications. The reasons for emergency department admissions, re-hospitalization and surgical requirements, time relationships, and possible antecedents were examined in stages.

**Results:**

A total of 153 patients were evaluated in the study. The average follow-up period was found to be 609.63 ± 222.89. The emergency department admission rate following MBSs was found to be 31%, and the admission rate within the first month was 7.8%. The major complication rate following MBSs was 4.6%. Experiencing complications was the most important antecedent for admissions 1 month postoperatively.

**Conclusions:**

In patients with complications after MBS, the discharge decision should be provided with stricter controls, and outpatient clinic controls should be planned more frequently. Providing training to patients on managing complications should be considered as a strategy that may reduce the number of emergency department visits.

**Clinical Trial Registration:**

ACTRN12624000810516

## Introduction

Obesity is associated with many medical problems, including Obstructive Sleep Apnea (OSA) [1] and type II diabetes, cardiovascular diseases, and cancer [2]. The indication threshold for MBS was reduced from BMI ≥ 40 kg/m^2^ to BMI ≥ 35 kg/m^2^ and in the presence of obesity-related medical problems, from BMI ≥ 35 kg/m^2^ to BMI ≥ 30 kg/m^2^ [3]. It is expected that the number of postoperative emergency room visits will increase in parallel with the development of laparoscopic techniques and the increasing prevalence of obesity in the society.

Complications and hospital readmission rates are important quality measures for surgical clinics [4]. The Clavien-Dindo Classification (CDC) has been preferred in clinics in recent years for the evaluation and reporting of postoperative complications in general surgery [5, 6]. The data on the use of CDC for MBS are limited. MBS can be considered a relatively safe surgery with low major complication rates [7]. A previous study reported the 30-day complication rate following laparoscopic sleeve gastrectomy (LSG) as 15.3% [8]. A different study that compared the 30-day morbidity and mortality of sleeve gastrectomy (SG), Roux-en-Y Gastric Bypass (RYGB), and one anastomosis gastric bypass (OAGB) reported that the difference in surgery type did not cause any significant differences in complication rates [7]. Anastomotic leakage, bleeding, anastomotic stenosis, stomach erosion, and small intestine obstruction are surgery-related complications that might be detected following MBSs [9]. A previous multicenter study reported the 30-day readmission rate as 4.4% [10].

Identification of patients who are at high risk for complications by surgical teams and informing patients and their relatives about this can prevent unnecessary emergency room visits. Emergency service professionals’ familiarity with the post-bariatric process might contribute to the targeted use of examination and imaging methods and can provide an increase in patient satisfaction and savings in the use of hospital resources. The present study aimed to determine the rates, characteristics, and antecedents of emergency department admissions within 2 years following MBSs performed in our hospital.

## Material and Methods

The present study was designed in a retrospective, observational, and single-center design. The study was evaluated at the meeting of Karamanoğlu Mehmetbey University Faculty of Medicine Ethics Committee on 20 June 2023. Ethical approval was received (Decision No: 06–2023/21) and was registered to ANZCTR on 01 July 2024 (ACTRN12624000810516). Necessary permissions were obtained to conduct the study in our hospital. The study was conducted in line with the Declaration of Helsinki. Individuals who underwent surgery were informed that their data could be used for scientific purposes during the preoperative period.

### Surgical Techniques

Primary surgeries were SG, gastric plication (GP), RYGB, OAGB, and revision bariatric surgeries (RBSs); bariatric conversions (BCs), RYGB, and OAGB were determined according to the patient’s clinical characteristics and the surgeon’s decision. All procedures were performed laparoscopically by one single surgeon under general anesthesia. If there are no complications after MBS in our clinic, those who have undergone SG and GP operations are monitored in the hospital for 2 days, and those who have undergone RYGB, OAGB, and RBS operations are monitored in the hospital for 4 days.

The nutritionist is a member of our multidisciplinary team and makes preoperative and postoperative evaluations; plans the patient’s nutrition in line with the surgery, starting from the beginning of oral intake in the postoperative period; and follows implementation.

### Inclusion Criteria

Individuals who were between the ages of 18 and 65 who had MBS and were classified according to CDC at their first postoperative follow-ups were included in the study.

### Exclusion Criteria

Patients whose data could not be accessed in the electronic database, patients diagnosed with psychiatric diseases that were not in remission, and those with a follow-up period of less than 3 month were excluded from the study.

### Data Sources

The data on the individuals who underwent MBS between June 2021 and June 2023 were evaluated from the hospital’s electronic database and from follow-up forms kept with patient consent for the follow-up of obese individuals who routinely undergo MBS in our hospital. The database provided data on patient-level demographics, diagnoses, surgical procedures, and hospital emergency department admission dates. The evaluation of complications for the first month was made by providing electronic database support with the Clavien-Dindo Classification System (CDC) as a treatment-based system used to rate complications in seven classes (I, II, IIIa, IIIb, IVa, IVb, and V) [11]. In the present study, CDC > III was considered a major complication. CDC is recorded online on the system in the first postoperative month for individuals with obesity and who undergo bariatric surgery. Records for the study were scanned retrospectively through the system.

### Study Variables

Age, gender, height, weight, and body mass index (BMI) values recorded in the preoperative anesthesia examination of individuals with obesity, for whom a surgery decision was made following the evaluation of a multidisciplinary team including general surgery, anesthesia, endocrinology, nutritionist, and psychiatry specialists, and hemoglobin (Hb) values measured with an automatic hematology analyzer and medical problems diagnosed at least 1 year before bariatric surgery were determined. Diabetes mellitus, hypertension, OSA diagnoses, preoperative Hb values, and CDC classifications were accepted as study variables. The presence of OSA was established by questioning whether the participants had been diagnosed before, based on the patient’s declaration. Smoking 10 or more cigarettes a day for more than 1 year was considered “smoking.” To be evaluated in statistical analysis, all morbidities of individuals undergoing bariatric surgery were categorized as Yes/No.

The emergency service visits of the patients following discharge were scanned in the database. The data on the first emergency department visits were recorded. Applications associated with upper respiratory tract infections, urinary tract infections, and pregnancy were not considered emergency applications associated with surgery. Complaints that caused postoperative emergency room visits were recorded as expressed by the patients. Primary diagnoses of outpatients who did not require hospitalization were obtained from the emergency department epicrisis reports. Secondary diagnoses of patients requiring hospitalization were obtained from the surgical service epicrisis reports during the period they were followed in the general surgery ward. The surgical requirements of the patients were queried through the system.

### Statistical Evaluation

Mean and standard deviation for numerical variables and frequency and percentage values for categorical variables were given as descriptive statistics. The chi-square and Fisher’s exact tests were used in the analysis of the categorical variables. The Mann–Whitney *U* and Kruskal–Wallis tests were used in the analysis of the numerical variables. Multivariate Cox regression analyses were used to find the variables that were associated with emergency admissions. The data analysis was performed with the R 4.3.2 program. *p* < 0.05 was considered significant.

## Results

A total of 153 patients suffering from MBS were evaluated in the present study (109 (71%) women). The average age was 39.85 ± 10.58, the average follow-up period of the sample was 609.63 ± 222.89, 140 patients (92%) had a follow-up period of 9 months to 2 years, 13 patients (8%) had a follow-up period of 3 months to 9 months, and 31% of the patients applied to the emergency department at least once during the follow-up period. The demographic and clinical characteristics of the patients who underwent MBS are given in Table 1.

**Table 1:** Demographic and clinical characteristics of patients undergoing metabolic and bariatric surgery

Variable	*N* = 153^a^
Sex	
Male	44 (29%)
Female	109 (71%)
Age(years)	39.85 ± 10.58
HT	
None	135 (88%)
Yes	18 (12%)
DM	
None	127 (83%)
Yes	26 (17%)
Preoperative hemoglobin, (g/L)	13.50 ± 1.82
Smoking status	
None	99 (65%)
Yes	53 (35%)
BMI (kg/m^2^)	43.34 ± 7.83
OSA	
None	127 (83%)
Yes	26 (17%)
Operation type	
Primer surgery	130 (84.9%)
RBS	23 (15.1%)
Emergency service application	
None	106 (69%)
Yes	46 (31%)
Hospitalization requirement	
None	130 (84.9%)
Yes	23 (15.1%)
Surgical application	
None	138 (90%)
Yes	15 (9.8%)

*HT* hypertension, *DM* diabetes mellitus, *BMI* body mass index (kg/m^2^), *OSA* obstructive sleep apnea, *RBS* revision bariatric surgeries

^a^n (%); mean ± SD; median (25–75%)

In the present study, the number of patients who underwent primary surgery was 130 (84.9%), and the number of patients who underwent revision bariatric surgeries (RBSs) was 23 (15.1%) (Table 1). The number of patients who visited to the emergency department after primary surgery was 36 (28.5%). The number of patients who visited to the emergency department after RBS was 10 (43.5%).

The CDC classification data of the patients and the times of admission to the emergency department in the first postoperative month after MBS in our clinic are given in Table 2. According to the CDC determined at the end of the first postoperative month, 78% of the patients who had MBS did not encounter any complications; 4.6% of individuals had MBS required surgical, endoscopic, and radiological interventions, which we consider as major complications. The rate of emergency department visits in individuals with MBS was 31% and 7.8% made their first emergency visits within the first month after surgery.

**Table 2:** Clavien-Dindo Classification System (CDC) classification of patients and times of admission to the emergency department in the first postoperative month after MBS

Variable	*N* = 153^a^
CDC	
O	120 (78%)
I	16 (10%)
II	10 (6.5%)
III +	7 (4.6%)
Time of admission to the emergency department	
Number of emergency department visits in the first postoperative month	12 (7.8%)
Postoperative 1–3 number of emergency department admissions in the month	7 (4.6%)
Postoperative 3–9 number of emergency department admissions in the month	15 (9.8%)
Number of emergency department admissions after ninth postoperative month	119 (78%)

*CDC* Clavien-Dindo Classification System

^a^n (%); mean ± SD; median (25–75%)

The complaints of those who applied to the emergency department following MBS are given in Table 3. The most common presentation was for abdominal pain. Nausea-vomiting and fatigue were the other most common complaints.

**Table 3:** Complaints at presentation to the emergency department after metabolic and bariatric surgery

Variable	*N* = 153^a^
Complaints for applying to the emergency department	
Nausea-vomiting	4 (2.6%)
Drain site bleeding	1 (0.7%)
Numbness in hands and feet	1 (0.7%)
Fatigue	3 (2.0%)
Abdominal pain	27 (18%)
Abdominal pain, nausea	1 (0.7%)
Abdominal pain, constipation	1 (0.7%)
Abdominal pain, vomiting	3 (2.0%)
Abdominal pain, discharge at the wound site	1 (0.7%)
Vomiting	2 (1.3%)
Anal bleeding, constipation	2 (1.3%)

^a^*n* (%); mean ± SD; median (25–75%)

Hospitalization diagnoses, hospitalization, and surgical requirements of the patients who were admitted to the emergency department and readmitted to the hospital following MBSs are given in Table 4. The most common hospitalization diagnosis was alkaline reflux gastritis (33%) in our study sample. Gastroesophageal reflux (GER) with 15% and dehydration with 13% were recorded as the other most common hospitalization diagnoses. Following discharge, 15% of the patients required re-hospitalization and applied to the emergency department, and 9.8% required surgical procedures following emergency admission.

**Table 4:** Hospitalization diagnoses for patients admitted to the emergency department and all MBSs

Variable	Patients admitted to the emergency department *N* = 46^a^	Total MBS *N* = 153^a^
Hospitalization diagnoses		
Anastomotic stenosis	1 (2.2%)	1 (0.6%)
Anastomotic leak	3 (6.5%)	3 (1.9%)
Intraabdominal abscess	2 (4.3%)	2 (1.3%)
Dehydration	6 (13%)	6 (3.9%)
Bleeding at the drain site	1 (2.2%)	1 (0.6%)
Alkaline reflux gastritis	15 (33%)	15 (9.8%)
GER	7 (15%)	7 (4.5%)
Hemorrhoid	2 (4.3%)	2 (1.3%)
Hernia	1 (2.2%)	1 (0.6%)
Ileus	2 (4.3%)	2 (1.3%)
Cholecystitis	5 (11%)	5 (3.2%)
Margınal ulcer	1 (2.2%)	1 (0.6%)

*GER* gastroesophageal reflux

^a^*n* (%); mean ± SD; median (25–75%)

The comparison of the demographic data and emergency admissions requiring hospital readmission is given in Table 5, and the comparison of emergency admissions requiring surgery is given in Table 6. A statistically significant relationship was detected between the presence of HT, low BMI, and the presence of complications at all times and the need for rehospitalization and surgery (Tables 5 and 6).

**Table 5:** Comparison of hospital readmission status after emergency department admissions and demographic data and study variables

Variable	None, *N* = 130^a^	Yes, *N* = 23^a^	*p* value
Sex			0.76
Male	38 (29%)	6 (26%)	
Female	92 (71%)	17 (74%)	
Age(years)	39.17 ± 10.29	43.70 ± 11.60	0.074
HT			0.007*
None	119 (92%)	16 (70%)	
Yes	11 (8.5%)	7 (30%)	
DM			0.074
None	111 (85%)	16 (70%)	
Yes	19 (15%)	7 (30%)	
Preoperative hemoglobin, (g/L)	13.54 ± 1.79	13.22 ± 2.01	0.46
Smoking status			0.75
None	84 (65%)	15 (68%)	
Yes	46 (35%)	7 (32%)	
BMI (kg/m^2^)	43.91 ± 7.52	40.16 ± 8.91	0.049*
OSA			0.37
None	106 (82%)	21 (91%)	
Yes	24 (18%)	2 (8.7%)	
CDC			< 0.001*
O	108 (83%)	12 (52%)	
I	15 (12%)	1 (4.3%)	
II	7 (5.4%)	3 (13%)	
III +	0 (0%)	7 (30%)	

*HT* hypertension, *DM* diabetes mellitus, *BMI* Body mass index (kg/m^2^), *OSA* obstructive sleep apnea

^*^p value is below the threshold of < 0.05

^a^n (%); mean ± SD; median (25–75%)

**Table 6:** Comparison of demographic data and study variables with the need for surgery after emergency department admissions

Variable	None, *N* = 138a	Yes, *N* = 15a	*p* value^b^
Sex			0.77
Male	39 (28%)	5 (33%)	
Female	99 (72%)	10 (67%)	
Age (years)	39.32 ± 10.23	44.73 ± 12.79	0.10
HT			0.003*
None	126 (91%)	9 (60%)	
Yes	12 (8.7%)	6 (40%)	
DM			0.14
None	117 (85%)	10 (67%)	
Yes	21 (15%)	5 (33%)	
Preoperative hemoglobin, (g/L)	13.46 ± 1.78	13.83 ± 2.23	0.26
Smoking status			0.56
None	91 (66%)	8 (57%)	
Yes	47 (34%)	6 (43%)	
BMI (kg/m^2^)	43.80 ± 7.72	39.19 ± 7.88	0.034*
OSA			> 0.99
None	114 (83%)	13 (87%)	
Yes	24 (17%)	2 (13%)	
CDC			< 0.001*
O	113 (82%)	7 (47%)	
I	15 (11%)	1 (6.7%)	
II	10 (7.2%)	0 (0%)	
III +	0 (0%)	7 (47%)	

*HT* hypertension, *DM* diabetes mellitus, *BMI* body mass index (kg/m^2^), *OSA* obstructive sleep apnea

^*^P value is below the threshold of < 0.05

^a^*n* (%); mean ± SD; median (25–75%)

^b^Fisher’s exact test; Wilcoxon rank sum test; Pearson’s chi-squared test

The duration of emergency admission was compared with the demographic and clinical characteristics separately for the first postoperative month, postoperative 1 to 3 months, postoperative 3–9 months, postoperative 9 months, and 2-year periods (Table 7). A statistically significant relationship was detected between age, the presence of HT and DM, the presence of smoking status, and complications.

**Table 7:** Comparison of demographic and clinical characteristics and duration of presentation to the emergency department

Variable	1 month, *N* = 12^a^	1–3 months, *N* = 7^a^	3–9 months, *N* = 15^a^	9 months + , *N* = 119^a^	*p* value^b^
Sex					0.79
Male	3 (25%)	3 (43%)	5 (33%)	33 (28%)	
Female	9 (75%)	4 (57%)	10 (67%)	86 (72%)	
Age (years)	46.83 ± 9.96	39.00 ± 9.50	44.53 ± 11.27	38.61 ± 10.30	0.024*
HT					0.015*
None	7 (58%)	6 (86%)	13 (87%)	109 (92%)	
Yes	5 (42%)	1 (14%)	2 (13%)	10 (8.4%)	
DM					0.007*
None	7 (58%)	5 (71%)	10 (67%)	105 (88%)	
Yes	5 (42%)	2 (29%)	5 (33%)	14 (12%)	
Preoperative hemoglobin, (g/L)	12.83 ± 1.94	14.37 ± 1.79	13.86 ± 1.93	13.46 ± 1.80	0.43
Smoking status					0.044*
None	9 (75%)	3 (43%)	5 (36%)	82 (69%)	
Yes	3 (25%)	4 (57%)	9 (64%)	37 (31%)	
BMI (kg/m^2^)	39.43 ± 10.29	43.33 ± 9.97	40.94 ± 10.17	44.04 ± 7.00	0.42
OSA					0.75
None	10 (83%)	6 (86%)	11 (73%)	100 (84%)	
Yes	2 (17%)	1 (14%)	4 (27%)	19 (16%)	
CDC					< 0.001*
O	6 (50%)	3 (43%)	14 (93%)	97 (82%)	
I	0 (0%)	1 (14%)	0 (0%)	15 (13%)	
II	1 (8.3%)	1 (14%)	1 (6.7%)	7 (5.9%)	
III +	5 (42%)	2 (29%)	0 (0%)	0 (0%)	

*HT* hypertension, *DM* diabetes mellitus, *BMI* body mass index (kg/m^2^), *OSA* obstructive sleep apnea

^*^P value is below the threshold of < 0.05

^a^*n* (%); mean ± SD; median (25–75%)

^b^Fisher’s exact test; Kruskal–Wallis rank sum test

Since the CDC classification is evaluated at the end of the 30th postoperative day in obese individuals, multivariate Cox regression analyses (Table 8) were performed by excluding the first 30-day admissions for the variables and are given in Table 8. A statistically significant relationship was detected between experiencing complications and seeking emergency admission according to age, presence of HT, and DM.

**Table 8:** Multivariate Cox regression analyses for the duration of emergency department visits and emergency department visits after the first postoperative month

Variable	HR	95% CI	*p*
Age (years)	1.02	0.99, 1.06	0.2
HT			0.6
None	—	—	
Yes	0.74	0.21, 2.61	
DM			0.2
None	—	—	
Yes	1.96	0.68, 5.68	
CDC			0.007*
O	—	—	
I	1.42	0.53, 3.82	
II	2.02	0.58, 7.04	
III +	55.1	9.60, 317	

*HR* hazard ratio, *CI* confidence interval, *CDC* Clavien-Dindo Classification System, *HT* hypertension, *DM* diabetes mellitus, *BMI* body mass index (kg/m^2^), *OSA* obstructive sleep apnea

^*^P value is below the threshold of < 0.05

## Discussion

It was found that 31% of the individuals who underwent MBS were admitted to the emergency department, with an average follow-up period of 609.63 ± 222.89 days. The fact that only 9.8% of these applications were within the first postoperative month shows that emergency department applications following MBSs might occur throughout the following period. We argue that experiencing postoperative complications is the most important determinant of emergency department admissions.

Patients who experience early complications after MBS can be treated in the hospital before their discharge, but they might present to the emergency department with acute abdominal pain that occurs months or years after surgery [12]. The mean follow-up period was 609.63 ± 222.89 days in the present study. Among the 153 patients who were included in the present study, 140 were followed up between 9 months and 2 years. When demographic and clinical data and emergency department application times were compared (Table 7), it was found that 119 patients presented to the emergency department at or after the ninth postoperative month. We also evaluated the effects of the complication status assessed at the end of the first postoperative month with the Clavien-Dindo Classification (CDC) on emergency department applications after the first postoperative month. Minor complications were defined only for the first month in the present study. We found that 16 patients who presented to the emergency department after the first month postoperatively without complications (CDC 0) or with minor complications (CDC I, CDC II) were readmitted to the hospital after presentation, and eight underwent surgery. Late or long-term complications (after the postoperative first month) resulting from MBS were not fully understood because of the variety of surgical procedures [12]. In a multicenter study, the emergency department presentation rate for MBS after the sixth month was reported to be 41.2% [12]. For example, anastomotic stenosis is seen in approximately 12% of patients after bypass and typically develops 1 month or more after surgery. It is most frequently detected 50 days after gastric bypass [9]. Similar evaluations can be made for marginal ulcers and small bowel obstruction (SBO). Marginal ulcer complications can occur in the early or late period [13]. In a previous study, the average presentation time for SBO after gastric bypass (GB) was reported to be 313 days [14]. Patients with uncomplicated or minor complications might present to the emergency department with clinical conditions that require urgent surgery in the long term.

One of the aims of the present study was to investigate the relationship between late emergency department visits after the first month postoperatively and patient demographic and clinical data. To this end, the relationship between the variables of diabetes mellitus (DM), hypertension (HT), preoperative Hb value, obstructive sleep apnea (OSA), smoking status, and complications in the first month postoperatively and emergency visits was evaluated. Smoking patients constituted a large proportion of our sample size (35%). Yüce et al. found that smokers were associated with more frequent rehospitalization, mortality, serious morbidity, wound complications, and respiratory complications in their study that evaluated bariatric surgeries [15]. Previous studies have evaluated that smoking status is associated with the development of marginal ulcers requiring surgical revision after MBS [16, 17]. We believe that it is important to evaluate the relationship between smoking status, which is a modifiable risk factor, and MBS results.

The relationship between the presence of HT, DM, and OSA, which are considered obesity-related medical problems; preoperative Hb value and smoking status; and emergency department visits in individuals with obesity was evaluated. Interestingly, no statistically significant relationships were detected between OSA and emergency admissions following emergency admissions. We think that the length of our follow-up period and the regression that might be detected in OSA cases following MBS are effective in our results.

Postoperative bleeding is a relatively common complication and might cause mortality and morbidity [18]. Although its contribution to the complications could not be determined, no statistically significant relationship was detected between postoperative emergency admissions and preoperative Hb values in the study.

A statistically significant relationship was detected between age, presence of HT, presence of DM, smoking status, and experiencing complications in the evaluation of emergency department visits over time; however, in the statistical evaluations, it was found that experiencing postoperative complications was the strongest determinant.

In the present study, it was also found that readmission was considered necessary following emergency department admissions in 15% of cases following MBS, and surgical intervention was required in 9.8%. The three most common complaints in these applications were abdominal pain, nausea-vomiting, and fatigue. The most common hospitalization diagnoses following emergency department admissions were alkaline reflux gastritis, GER, and dehydration. Tuğcan et al. reported a 22% rate of emergency department visits in the patient population that were followed for 6 months following bariatric surgery [19]. The most common complaint in emergency department admissions was abdominal pain, and the most common diagnosis in patients deemed suitable for hospitalization was perforation [19]. We think that the long follow-up period in the present study and the large number of patients whose first admission to the emergency department was following the ninth postoperative month were the reasons for the difference in hospitalization diagnoses. In their study that reported their 1-year follow-up data following bariatric surgery, Pinzon et al. found the frequency of admission to the emergency department between 10.7% (postoperative days 0–30) and 5.7% (postoperative days 181–270) [20]. We think that long-term complications of bariatric surgery, e.g., cholecystitis [21] and marginal ulcer [22] among the diagnoses of rehospitalization after emergency admissions, were also reflected in our results because of the large number of follow-ups up to 2 years. We argue that information on how to manage abdominal pain following MBSs must be given to patients who will undergo MBS in the preoperative period and to emergency department and surgical team staff as part of their in-service training.

A total of 78% of our patients completed their first postoperative month without any complications; 4.6% faced major complications that required surgical, endoscopic and radiological interventions. In the present study, the early-period (postoperative first month) major complication rate after MBS was defined as patients who were graded CDC III and higher. The early-period major complication rate (as seen in Table 2) was 4.6% at the end of the first month postoperatively. Davey et al. examined five RCTs in their meta-analysis and determined a major complication rate of 3.4% [23]. Our leakage rate was found to be 1.9% after MBS at all times and 6.5% among patients who required hospitalization after emergency room visits. De Simone et al. [12] reported leakage rates of 4.2% in their study. Abdegavad et al. published their 3-year follow-up results in RBS and examined 81 RBSs reporting eight major complications (10.4%), five of which were leaks [24]. The emergency visit rate after RBS was found to be 43.5% in the present study. We think that the high complication and leakage rates in the present study were associated with the presence of revision bariatric surgeries (RBSs) performed at a rate of 15.1% in our sample (Table 1). Our high complication survival rates might have occurred because of the presence of RBSs in our sample. RBS is technically more complex than primary MBS and is associated with increased hospital stay and higher complication rates [24–26]. We hope that studies in which different types of surgeries will be performed separately will be published in the future after the appropriate sample size is reached.

In the present study, nearly half of the patients who applied to the emergency department required hospitalization, which was higher than previous studies reporting results on this subject. In their study, Telem et al. reported that 34.9% of the patients [27] and Pinzon et al. reported that approximately one-fourth of the patients were readmitted following emergency department admissions following admission to the emergency departments [20]. Our higher readmission rates might be associated with the presence of RBS in our samples and the wider use of hospitalization indications, considering the transportation difficulties of patients who lived outside the city.

When the hospital readmission and surgical requirements that were evaluated in our study were compared with demographic data and clinical characteristics, the presence of HT was detected, and lower BMI values and higher CDC values were significant for hospital readmission and surgical necessity. In our study, the researchers found the need for readmission and surgery to be associated with lower BMI. The reason for this might be RBSs in our sample size. Bariatric conversions are the most common RBS procedure [28]. The incidence of RBS is estimated to be between 5 and 26% [29]. The most common causes of RBS are stomach acid reflux, bile reflux, fistula, leak, unexplained abdominal pain, protein-calorie malnutrition (PCM), and stricture [28]. We think that PMC-associated low BMI values affected the need for re-hospitalization and surgery following emergency admission in our study.

One of our limitations is that our study was conducted in a single center and by a single surgeon. The fact that the studies were conducted retrospectively in one single center and with a long follow-up period carries the possibility of missing data. There is a possibility of emergency service applications to different centers. We consider this as the limitation of the present study. We tried to minimize this by screening our patient inquiries during repeated outpatient clinic checks associated with our long follow-up period. Our sample size was heterogeneous for surgical procedures, and this heterogeneity might have different reasons for admission in the postoperative period; however, only the main presenting complaints and diagnoses could be recorded nonspecifically. We think that prospective studies that can eliminate these limitations will be added to the literature in the future.

## Conclusion

In the present study, it was shown that emergency department visits following MBS continued significantly after 1 month postoperatively and that advanced age, hypertension, DM, smoking, and experiencing postoperative complications were effective in emergency department visits. The researchers presented the data showing that the most important determinant among all these factors is experiencing postoperative complications.

## Data Availability

The data used and/or analyzed in the present study are available from the corresponding author upon reasonable request.
